# The Role of ADHD Genetic Propensity in Dementia Risk

**DOI:** 10.1002/alz70855_103947

**Published:** 2025-12-23

**Authors:** Yannick Joel Wadop Ngouongo, Bernard Fongang, Stephen Z Levine, Galit Weinstein

**Affiliations:** ^1^ Glenn Biggs Institute for Neurodegenerative Diseases, University of Texas Health Science Center at San Antonio, San Antonio, TX, USA; ^2^ Glenn Biggs Institute for Alzheimer's & Neurodegenerative Diseases, The University of Texas Health Science Center at San Antonio, San Antonio, TX, USA; ^3^ Department of Population Health Sciences, The University of Texas Health Science Center at San Antonio, San Antonio, TX, USA; ^4^ Department of Biochemistry and Structural Biology, The University of Texas Health Science Center at San Antonio, San Antonio, TX, USA; ^5^ School of Public Health, University of Haifa, Haifa, Mount Carmel, Israel

## Abstract

**Background:**

Attention Deficit Hyperactivity Disorder (ADHD) may be related to adverse health behavior and increased risk of dementia in older ages. However, a substantial misclassification exists in ADHD clinical diagnosis due to its high prevalence yet extensive under‐diagnosis, particularly among individuals who are currently adults. To overcome this limitation, we explored the link between ADHD genetic polygenic risk scores (PRS) and incident dementia, as well as the role of lifestyle and health perception as potential mediators.

**Method:**

Utilizing data from the UK Biobank, ADHD PRS were computed using GWAS summary statistics. All‐cause dementia, vascular dementia (VaD), and Alzheimer's disease (AD) were ascertained via ICD codes. Associations were analyzed using multivariable linear and logistic regression models adjusted for age, sex, ethnicity, and population structure. Formal mediation analyses assessed the indirect effects of lifestyle and health perception on dementia outcomes.

**Result:**

Our cohort included 486,861 dementia‐free individuals with genetic information. Mean age was 56.5±8.1 years and 264,015 (54.2%) were women. Greater ADHD genetic propensity was significantly associated with higher risks of all‐cause dementia, VaD and AD (β=1.69E‐06, *p* = 0.00529; β=2.75E‐06, *p* =  0.03667 and β=1.79E‐06, *p* = 0.046175, respectively). Further, higher ADHD PRS was associated with increased odds for smoking and coffee consumption, but with higher odds for moderate physical activity and lower odds for using alcohol. Increase ADHD PRS was also significantly correlated with poorer overall health perception. Mediation analysis identified smoking as a significant mediator in the relationship between ADHD PRS and all‐cause dementia (proportion mediated = 7%, *p* < 0.05) and VaD (proportion mediated = 9.2%, *p* < 0.05), but not AD.

**Conclusion:**

This study provides evidence of a significant relationship between ADHD genetic propensity and dementia risk in middle‐aged and older adults and highlights the mediating role of smoking in this association. These findings underscore the importance of addressing modifiable lifestyle factors in mitigating dementia risk, particularly among individuals with higher genetic susceptibility to ADHD.

